# Assessing the role of ontogenetic movement in maintaining population structure in fish using otolith microchemistry

**DOI:** 10.1002/ece3.4186

**Published:** 2018-07-16

**Authors:** Peter J. Wright, Thomas Régnier, Fiona M. Gibb, Julian Augley, Sandhya Devalla

**Affiliations:** ^1^ Marine Scotland Science Aberdeen UK; ^2^ Present address: Fios Genomics Nine Edinburgh Bioquarter Edinburgh UK; ^3^ Present address: Environmental and Biochemical Sciences The James Hutton Institute Craigiebuckler Aberdeen UK

**Keywords:** Atlantic cod, marine fish, natal homing, otolith chemistry, philopatry, population structure

## Abstract

Identifying the mechanisms maintaining population structure in marine fish species with more than a single dispersing life stage is challenging because of the difficulty in tracking all life stages. Here, a two‐stage otolith microchemistry approach to examining life‐stage movement was adopted, tracking a year‐class from the juvenile to adult stage and inferring larval sources from clustering, in order to consider the mechanisms maintaining population structuring in North Sea cod. Clustering of near‐core chemistry identified four clusters, two of which had either a southern or northern affinity and were similar to juvenile edge chemistry. The other two clusters, common to the central North Sea, had intermediate chemical composition and may have reflected either larval mixing in this region or a lack of geographic heterogeneity in the elemental signature. From the comparison of whole juvenile and the corresponding component of adult otoliths, adults from the southern North Sea mostly recruited from adjacent nursery grounds. In contrast, many adults in the northern North Sea had a juvenile chemistry consistent with the Skagerrak and juveniles from the northern Skagerrak site had a near‐core chemistry consistent with the northern North Sea. Similarities in otolith chemistry were consistent with retention of early life stages at a regional level and also juvenile and adult fidelity. The links between the northern North Sea and Skagerrak indicate natal homing, which when considered in the context of genetic evidence is suggestive of philopatry. The approach used here should be useful in exploring the mechanisms underlying population structuring in other species with multiple dispersive life stages and calcified hard parts.

## 1 INTRODUCTION

The way in which animal populations are structured in space and time affects how they respond to environmental and human pressures (Schindler et al., 2010). Two key processes help shape and maintain population structure in the open seas: biogeographic barriers and philopatry. Oceanographic processes such as currents or upwelling that segregate planktonic offspring of different spawning aggregations and the availability of suitable habitat are the main biogeographic barriers (Miller, Versace, Matthews, Montgomery, & Bowie, 2013). Some species may also exhibit a form of philopatry, actively returning to their natal site or region as juveniles or adults (Bonanomi et al., 2016), which may lead to reproductive isolation. Within a generation, individuals may exhibit natal homing to the spawning area they originated from or adopt the migratory behavior of whichever adult group they settle to while originating from a common pool of propagules, that is, the adopted migrant hypothesis (McQuinn, 1997; Petitgas, Secor, McQuinn, Huse, & Lo, 2010). Consequently, an understanding of how population structure is maintained requires knowledge of dispersal among all life stages.

Testing for natal homing is very challenging as it requires the tracking of individuals from fertilization until reproduction (Bradbury & Laurel, 2007). Otolith chemistry has proved useful in examining movements among life stages in regions where there is detectable spatial variation (Campana & Thorrold, 2001). Although otolith chemistry is affected by water chemistry, physiology, and inheritance (Barnes, Gillanders, & Rose, 2013; Clarke, Thorrold, & Conover, 2011; Walther, Kingsford, O'Callaghan, & McCulloch, 2010), the resulting elemental “signature” can act as a natural tag (Campana & Thorrold, 2001; Elsdon et al., 2008). Unfortunately, as otolith chemistry can vary over time in relation to temperature, salinity, and ontogeny, as well as water mass occupied (Stanley et al., 2015; Walther et al., 2010), microchemistry profiles may not provide a reliable means of spatially tracking an individual. Therefore, connectivity can only be reliably estimated by comparing microchemistry in equivalent parts of the otolith from the same year‐class, thus allowing post‐dispersed individuals to be assigned to sampled sources (Gillanders, 2002; Munch & Clarke, 2008; Thorisson, Jónsdóttir, Marteinsdottir, & Campana, 2011; Wright, Neat, Gibb, Gibb, & Thordarson, 2006). Such an approach allows for the estimation of natal homing for those species whose larvae are restricted to estuaries or other confined water masses (Thorrold, Latkoczy, Swart, & Jones, 2001; Walther, Thorrold, & Olney, 2008). However, due to the potential for wide dispersal and high mortality, it is very difficult to track the movements of larvae using otolith chemistry. This has led some to infer the number of distinct natal sources and the spatial scale over which larval dispersal takes place from a cluster analysis of near‐core ablation chemistry (Calò et al., 2016; Gibb, Regnier, Donald, & Wright, 2017).

Atlantic cod, *Gadus morhua*, is well suited to studying the nature of population structuring because there is already considerable information on their movements (Neuenfeldt et al., 2013) and genetic differentiation (Bradbury et al., 2013), including evidence for natal fidelity (Barth et al., 2017; Bonanomi et al., 2016). In the North Sea, studies of microsatellite DNA have supported a degree of reproductive isolation between the deeper northeastern region between 100 and 200 m and shallower depths, although the neutrality of some key markers has been questioned (Hutchinson, Carvalho, & Rogers, 2001; Nielsen et al., 2009), and single‐nucleotide polymorphic markers under selection considerably improve the significance of these differences (Heath et al., 2014; Poulsen, Hemmer‐Hansen, Loeschcke, Carvalho, & Nielsen, 2011). At a finer spatial scale, analyses of otolith chemistry indicate that juvenile cod settling off the Scottish coast mainly recruit to their local spawning area (Wright, Neat, et al., 2006). At a similar scale, spawning fidelity has been indicated from tag recapture (Righton, Quayle, Hetherington, & Burt, 2007; Wright, Galley, Gibb, & Neat, 2006) and electronic tagging studies (Neat et al., 2014; Svedäng, André, Jonsson, Elfman, & Limburg, 2010). Physical segregation of cod larvae has been proposed from their distribution relative to frontal areas (Munk, Wright, & Pihl, 2002; Munk et al., 2009) and biophysical model evidence of retention in the northern North Sea (Heath, Kunzlik, Gallego, Holmes, & Wright, 2008), although some larvae are expected to be advected into the Skagerrak (Jonsson, Corell, André, Svedäng, & Moksnes, 2016). Hence, genetic and tagging evidence suggests that the North Sea stock comprises two or more populations: one inhabiting depths >100 m in the northern North Sea and another inhabiting the shallow southern shelf <50 m depth and possibly further coastal groups around the northern UK. Further, both biogeographic barriers and natal homing have been proposed as possible mechanisms shaping population structure in North Sea cod (Heath et al., 2008; Svedäng et al., 2010).

In this study, otolith chemistry was used to examine the role of retention, migrant adoption, and natal homing in shaping population structuring of North Sea cod. Laser ablation inductively coupled plasma mass spectrometry (LA‐ICP‐MS) was applied to one of the sagittal otoliths from recently settled juveniles to examine the contribution of segregated larval sources (Heath et al., 2008) to nursery grounds using a clustering approach (see Gibb et al., 2017). To estimate the extent of nursery to spawning ground movement, the other sagittal otolith was used in whole solution (WS‐ICP‐MS) to define a juvenile baseline for North Sea nursery grounds. Then, adult fish of the same year‐class sampled on known spawning grounds during the spawning season were assigned to these nursery grounds based on the chemical signature of the equivalent juvenile region of their otolith, also determined by WS‐ICP‐MS. The combination of these two approaches carried out on the same individuals gave insights on the roles played by the adopted migrants’ hypothesis or natal homing in shaping cod population structure in the North Sea. Finally, otolith evidence was reviewed in the context of reported genetic structuring to consider philopatry.

## 2 MATERIALS AND METHODS

### 2.1 Sample collection

**Figure 1:**
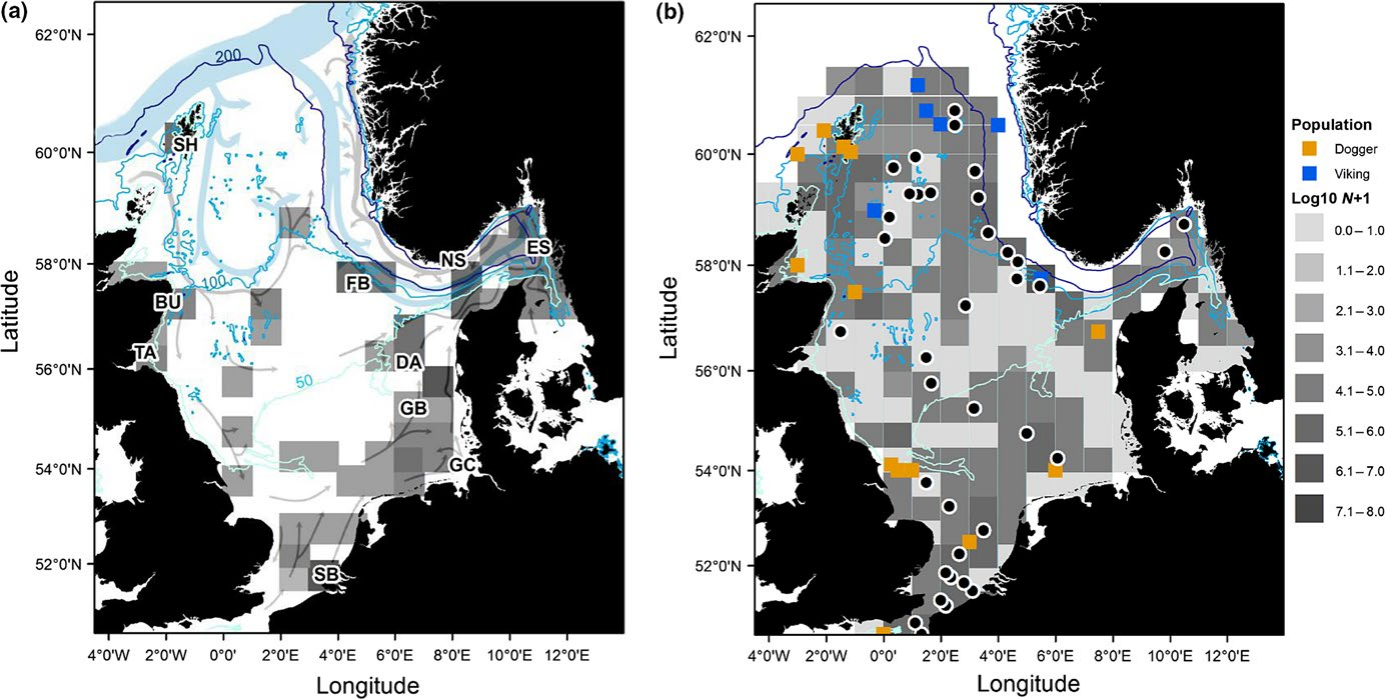
(a) Chart showing sample locations of 0‐group juveniles in autumn 2007 (site codes) overlaid on the density distribution of 0‐group in the IBTS survey (gray shading). Persistent North Sea circulation patterns are also shown, where blue arrows indicate North Atlantic inflow and gray arrows more coastal flow (OSPAR, 2000; after Turrell, Henderson, Slesser, Payne, & Adams, 1992). Sampled sites SB = Southern Bight, GB = German Bight, GC = German coast, DA = Danish coast, TA = Tay, BU = Buchan, ES = eastern Skagerrak, NS = northern Skagerrak, FB = Fisher Bank, SH = Shetland. (b) Chart showing adult sample locations in 2009 and 2010 (circles) overlaid on the density distribution of age 2 and 3 cod (gray shading). Locations of cod assigned to the Viking and Dogger population units (Heath et al., 2014; Poulsen et al., 2011) are also shown (squares), highlighting the segregation around depths >100 m in the northern North Sea.

Young of the year (0‐group), hereafter referred to as juvenile cod, were sampled for their sagittal otoliths in 2007 from areas covering the major concentrations and geographic extent of their distribution within the North Sea (Figure 1a). These densities have been overlaid on the sea circulation patterns persisting within the North Sea (Figure 1a). Juvenile samples were collected in autumn, just after the time when cod have settled to their demersal habitat (Bastrikin, Gallego, Millar, Priede, & Jones, 2014), from the International Council for the Exploration of the Sea (ICES) quarter 3 coordinated trawl surveys, dedicated Scottish research vessel and commercial charter vessel surveys. Sagittal otoliths from the corresponding year‐class of first‐time mature age 2 or 3 (sample age being dependent on region due to population differences in maturation onset; Wright, Millar, & Gibb, 2011) spawning cod were sampled during ICES International Bottom Trawl Surveys in quarter 1 of 2009 and 2010, respectively. A total of 431 juveniles were sampled from 10 sites (Figure 1a), and 442 age 2 or 3 adults were sampled from areas of high adult densities across the North Sea (Figure 1b). While there was a large range in temperature among sites (range 11.0–18.6°C), there was only a narrow salinity range (33.21–34.5).

### 2.2 Otolith preparation

Juvenile otoliths were decontaminated as per Gibb, Gibb, and Wright (2007). One sagittal otolith from all 431 juveniles collected was stored for WS‐ICP‐MS. The second sagittal otolith from 15 randomly selected juveniles from each site (except the German coast) was prepared for LA‐ICP‐MS (*n* = 135). Samples were randomized with respect to capture site to prevent sample batch bias before being mounted in epoxy resin (Araldite^®^ CY212, Agar Scientific) in groups of 5–6. Otoliths were sectioned transversely through the core using an Isomet^®^ 1000 precision linear saw (Buehler, Coventry, UK). The juvenile component of the otolith from spawning adults (age 2 or 3) was prepared for WS‐ICP‐MS. Otoliths were cleaned, embedded in epoxy resin, and sectioned as per the juveniles, before microsampling of the juvenile region using a New Wave MicroMill™, following the procedure of Charlier et al. (2006). The otolith was drilled to a depth of 500 μm over two passes (each pass to a depth of 250 μm) to remove most of the juvenile component, which was easily identifiable under reflected light. As a secondary measure to ensure that only material from the 0‐group part of the otolith was included, a threshold core length, width and depth limit were predefined electronically, set by averaging the cross‐sectional area of otoliths from a select group (*n* = 4) of 0‐group juveniles representing the lower size range (<10 cm TL).

### 2.3 Otolith elemental analysis

#### 2.3.1 Natal sources (near‐core LA‐ICP‐MS)

Juvenile otoliths were analyzed for the presence of eight isotopes ^26^Mg, ^43^Ca, ^55^Mn, ^65^Cu, ^66^Zn, ^86^Sr, ^88^Sr, and ^138^Ba, using a PerkinElmer Elan *DRCII+* ICP‐MS (PerkinElmer, Buckinghamshire, UK) equipped with a New Wave Research UP213 laser ablation system, using argon gas as the carrier. Pre‐ablation runs were undertaken on the standard and each otolith to remove any extraneous impurities. Material was ablated from two pits (55 μm diameter) on each otolith, one adjacent to, but not covering, the primordium (reflecting natal environment, at an average of 11 increments from the core) and one at the edge (reflecting local environment at capture). At the beginning, after each otolith and at the end of each experimental run of five to six otoliths, an argon gas blank and a glass standard reference material (NIST 612; National Institute of Standards and Technology) were taken for calibration and instrument drift correction. Blank subtracted count data were gathered for each sample ablation in Elan^®^ 3.4 software and converted to element concentrations (μg/g) in the otoliths by manual calculations using the internal standardization equation described by Longerich, Jackson, and Günther (1996), with concentrations of each element being determined relative to the NIST 612 standard. All isotopes were calculated as ratios relative to ^44^Ca, compensating for any variation in ablation yield between samples and standards.

#### 2.3.2 Juvenile baseline and corresponding component of adult otoliths (WS‐ICP‐MS)

The preparation of the whole juvenile otoliths and the material milled from the juvenile component of adult otoliths was designed to account for differences in sample size and weight. Juvenile whole otoliths were weighed to the nearest 0.001 mg and then digested in 1 ml 50% ultrahigh purity nitric acid (Seastar). Material from the juvenile component of the adult samples was also digested in a comparable acid, while maintaining the material ratio similar to that of the whole juvenile otoliths. To quantify potential contamination during the cleaning and preparation process, a series of blank acid‐washed vials and samples of resin were prepared in the same manner. All samples were randomly ordered with respect to location to prevent sample batch bias although due to the stage‐related difference in material analyzed, juvenile otoliths were run as different batches from the 2‐ and 3‐group otoliths. All element data were normalized as a ratio of calcium.

The elemental signature of the powdered otolith material from juvenile and the corresponding component of adult samples was analyzed using flow injection (FI) ICP‐MS. A PerkinElmer Sciex Elan 6100*DRC+* ICP‐MS equipped with an S10 autosampler, ultraclean autosampler probe (0.25 mm internal diameter), FIAS‐400MS FI system, PFA‐ST microconcentric nebulizer, and Peltier‐cooled cyclonic spray chamber (PC^3^, Elemental Scientific Inc., USA) were used for the analyses. Details of the ICP‐MS instrumentation and operating conditions are described in Robinson, Devalla, Rompais, and Davies (2009). Otolith isotope concentrations of ^26^Mg, ^55^Mn, ^88^Sr, and ^138^Ba were determined using ^72^Ge or ^103^Rh as internal standards. For quality control purposes, a procedural blank and accurately weighed otolith certified reference material (FEBS‐1; NRCC, Canada) were digested and analyzed with every 10 otolith samples.

### 2.4 Statistical analyses

As the data did not fully meet multivariate parametric assumptions of normality and homogeneity of variance/covariance, methods that relaxed or did not require these assumptions were used.

Differences in elemental ratios at the ablated otolith edge or in the whole otolith among juvenile sites were tested using Kruskal–Wallis and a Dunn multiple comparison test using the dunn.test package (v1.3.3) in R 3.3.1.

The potential for geographic variation in ablated edge chemistry to discriminate juveniles between sampling locations was assessed using random forest (RF) classification (Breiman, 2001), estimated with the randomForest package (v4.6.10) in R 3.3.1. The built‐in cross‐validation method of the RF approach (Out‐of‐Bag classification error; Breiman, 2001) was used to assess the accuracy with which sampling locations could be discriminated from each other (Gibb et al., 2017). Variable importance was assessed using the “mean decrease in accuracy”, a measure that reflects the loss in classification accuracy when a given variable is left out of the analysis (averaged across all trees; Breiman, 2001).

### 2.5 Sources of juveniles

Clustering analysis of the near‐core ablation data was performed to gain insights into the number of sources of juveniles and the movements of larvae between the locations sampled in this study. A RF clustering method was used in an unsupervised way, hereafter called RF clustering (Régnier et al., 2017). RF clustering is a two‐step process. In the first step, unsupervised RF was applied to the standardized dataset. Capture location (relevant for settled individuals) was ignored in this process; instead, a synthetic dataset was created through random sampling from the product of empirical marginal distributions of the different elements. Then, RF was used to separate observed from synthetic data (used as the class factor) and to produce a similarity matrix, defined by the frequency at which two individuals end up in the same terminal node of the trees (Breiman, 2001). In the second step, the dissimilarity matrix (dissimilarity = √(1 − similarity)) was used as an input for partitioning around medoid clustering and the appropriate number of clusters was determined using the Dunn index (see Régnier et al., 2017).

### 2.6 Adult assignment

The whole juvenile otolith chemistry, derived from WS‐ICP‐MS, was used as a baseline for assignment of the corresponding juvenile component of adult chemistry. The contribution of each baseline area to the adult samples was estimated from an assignment analysis in a Bayesian framework (or MCMC; Munch & Clarke, 2008). Here, the assumptions of strict multivariate normality and homogeneity of covariance matrices are relaxed by accounting for uncertainty in the baseline. Individuals were considered to be assigned to one of the 10 sites if the assignment probability was greater than an arbitrary threshold of 0.5.

## 3 RESULTS

### 3.1 Spatial variation in elemental signature of juveniles

**Figure 2:**
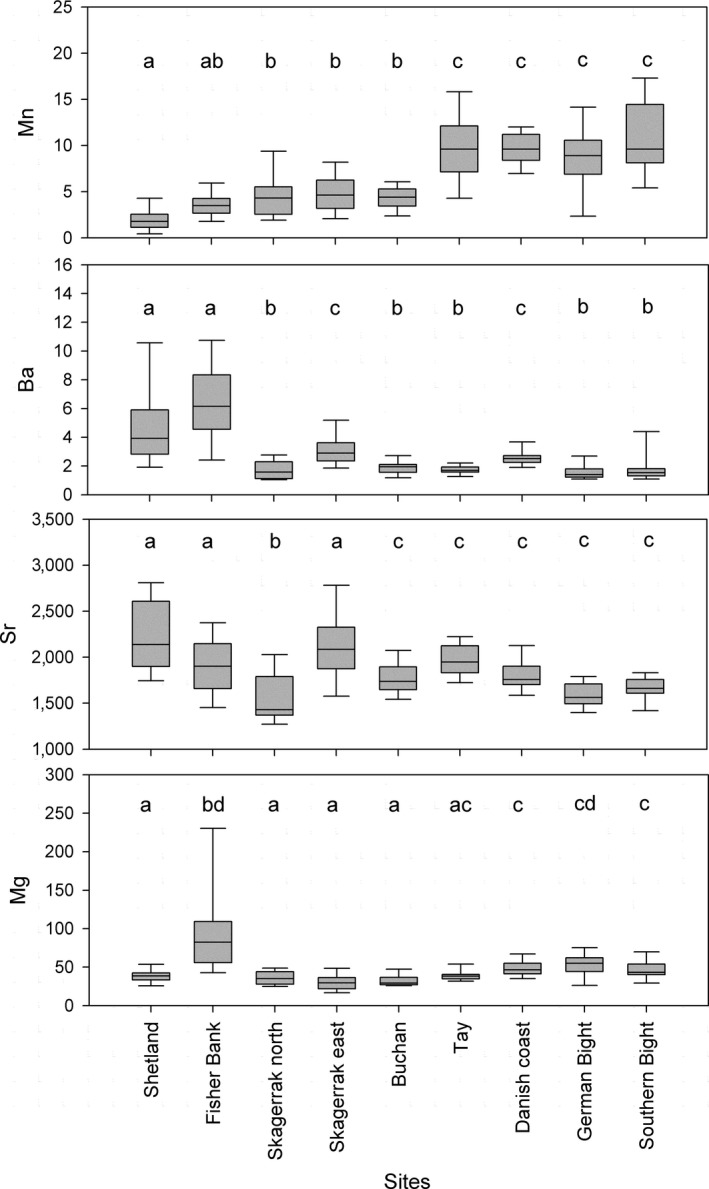
Box plots showing elemental variation, from LA‐ICP‐MS, for Mn, Ba, Sr, and Mg at the otolith edge (filled bars). The solid line corresponds to the median, the top and bottom of the box are the 75th and 25th percentiles, while the whiskers show the 5% and 95% percentiles, respectively. Different letters refer to significantly different sites based on Dunn's multiple comparison test (*p* < 0.05)

The ablated edge chemistry of the juvenile otolith showed significant geographic variation for Mn (*H* = 177.13, 8 *df*,* p* < 0.0001), Ba (*H* = 170.57, 8 *df*,* p* < 0.0001), Sr (*H* = 118.06, 8 *df*,* p* < 0.0001), and Mg (*H* = 129.54, 8 *df*,* p* < 0.0001). There was around a threefold range in median Mn, Mg, and Sr and a 1.6 times range in Ba among sites (Figure 2). Post hoc comparisons indicated significant differences between sites, with a negative latitudinal gradient in Mn (Spearman's rank correlation *r*_s_ = −0.72; *p* < 0.001) and a positive latitudinal gradient in Ba (*r*_s_ = 0.47; *p* < 0.001; *n* = 122). The northern sites of Shetland and Fisher Bank had high Ba, and the Tay and sites further south had high Mn. For Sr, smaller‐scale gradients were found with values declining from Shetland to Skagerrak and from the Tay to the southern North Sea. High Mg values were found in Fisher Bank and Danish coast which are both on the eastern side of the North Sea.

Differences in ablated edge elemental values among juvenile sites were reflected in the RF assignment. Classification accuracy by station ranged from 0.47 to 0.93, although most misassignments were to nearby grounds. From this, four regions of similar chemistry could be distinguished: northern sites (Shetland and Fisher Bank), Skagerrak sites (north and east), southern sites (Danish coast, German and Southern Bights), and off the Scottish east coast. Buchan, close to the 100‐m contour, differed from the more southerly Tay station which had an affinity with southern Dogger sites. There were no misassignments between the northern and southern sites (Table 1). Variable importance measured as the mean decrease in classification accuracy revealed that Mn was the most relevant element (24.5% mean decrease in classification accuracy), followed by Ba (16.1%), Sr (15%), and Mg (7.9%).

**Table 1:** Average cross‐validated classification probabilities of ablated otolith edge chemistry for juvenile sites (excluding the German coast; see Figure 1 for site codes) calculated on 100 independent random forests (derived from out‐of‐bag error rate). Shaded values refer to classification probability for site

Region	Northern		Skagerrak		East coast		Southern		
Site	SH	FB	NS	ES	BU	TA	DA	GB	SB
SH	0.87	0.00	0.00	0.13	0.00	0.00	0.00	0.00	0.00
FB	0.07	0.87	0.00	0.06	0.00	0.00	0.00	0.00	0.00
NS	0.00	0.00	0.93	0.00	0.07	0.00	0.00	0.00	0.00
ES	0.00	0.13	0.00	0.60	0.13	0.00	0.07	0.07	0.00
BU	0.00	0.07	0.07	0.07	0.73	0.00	0.00	0.06	0.00
TA	0.00	0.06	0.00	0.07	0.00	0.47	0.13	0.20	0.07
DA	0.00	0.00	0.00	0.00	0.07	0.20	0.47	0.13	0.13
GB	0.00	0.00	0.00	0.00	0.00	0.07	0.13	0.47	0.33
SB	0.00	0.00	0.00	0.00	0.00	0.00	0.07	0.26	0.67

### 3.2 Sources of juveniles

Although juveniles at a site could have originated from multiple sources, there was no significant difference in Ba, Mn, and Mg between the ablated near‐core and edge chemistry in eastern Skagerrak juveniles or for Ba and Mn in Buchan juveniles (Wilcoxon's matched‐pair test; *p* > 0.1; Table 2). Ba levels were also consistently high between the near core and edge of northern sites (Shetland and Fisher Bank) and conversely consistently low in the more southern Danish coast juveniles. Near‐core Ba levels of north Skagerrak juveniles were also significantly higher than those in the edge (Wilcoxon's matched‐pair test; *p* < 0.001) and similar to the high levels found on the otolith edge of the juveniles of Shetland and Fisher Bank.

**Table 2:** Comparison between ablated edge and near‐core element values by site for four elements based on *p*‐values from Wilcoxon's matched‐pair test

Site	Mn	Ba	Sr	Mg
SH	0.001	0.561	0.000	0.095
FB	0.000	0.804	0.041	0.135
NS	0.000	0.000	0.035	0.010
ES	0.095	0.978	0.000	0.121
BU	0.135	0.303	0.015	0.010
TA	0.018	0.003	0.048	0.303
DA	0.002	0.720	0.002	0.001
GB	0.001	0.022	0.035	0.000
SB	0.000	0.001	0.001	0.000

**Figure 3:**
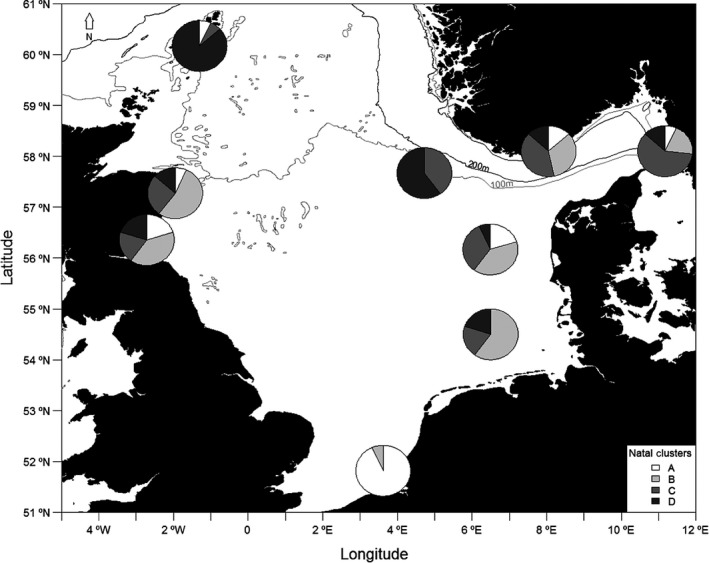
Pie charts representing the contribution of the four natal sources (A‐D), identified through RF clustering of ablated near‐core chemistry, to settled juvenile sites

The RF clustering of near‐core ablation chemistry distinguished between four and seven natal clusters based on early larval elemental values. Given the four regions of similar edge chemistry, the four larval cluster solutions were considered further. The mean and standard deviation of each element value per cluster are given in Table 3. Cluster A, characterized by low Ba and high Mg, Mn, and Sr, was the main contributor to Southern Bight and was absent from German Bight and Fisher Bank (Figure 3). Cluster B, characterized by low Ba and high Mn, was the main contributor to German Bight, Danish coast, Tay, and Buchan but was not found in Shetland or Fisher Bank. Cluster C, characterized by low Ba, Mn, Sr, and Mg, contributed most to Skagerrak samples but also to Fisher Bank. Cluster D, characterized by high Ba and low Mn, was the main contributor to Shetland and Fisher Bank and was absent in Southern Bight.

**Table 3:** Mean element values for the four natal clusters identified by RF clustering; standard deviation is indicated between brackets

Cluster	Element value			
	Mg	Mn	Ba	Sr
A	85.9 (23.4)	5.2 (2.7)	4 (2.3)	2,031.4 (433.7)
B	70.2 (11.8)	6.1 (3.3)	2.9 (1.7)	1,867 (497.3)
C	66 (14.4)	3.1 (1.6)	2.8 (1.2)	1,474.5 (222.3)
D	77.5 (17.6)	3.6 (4.5)	7.9 (4)	2,013.8 (429)

### 3.3 Spatial variation in whole‐solution juvenile chemistry

**Figure 4:**
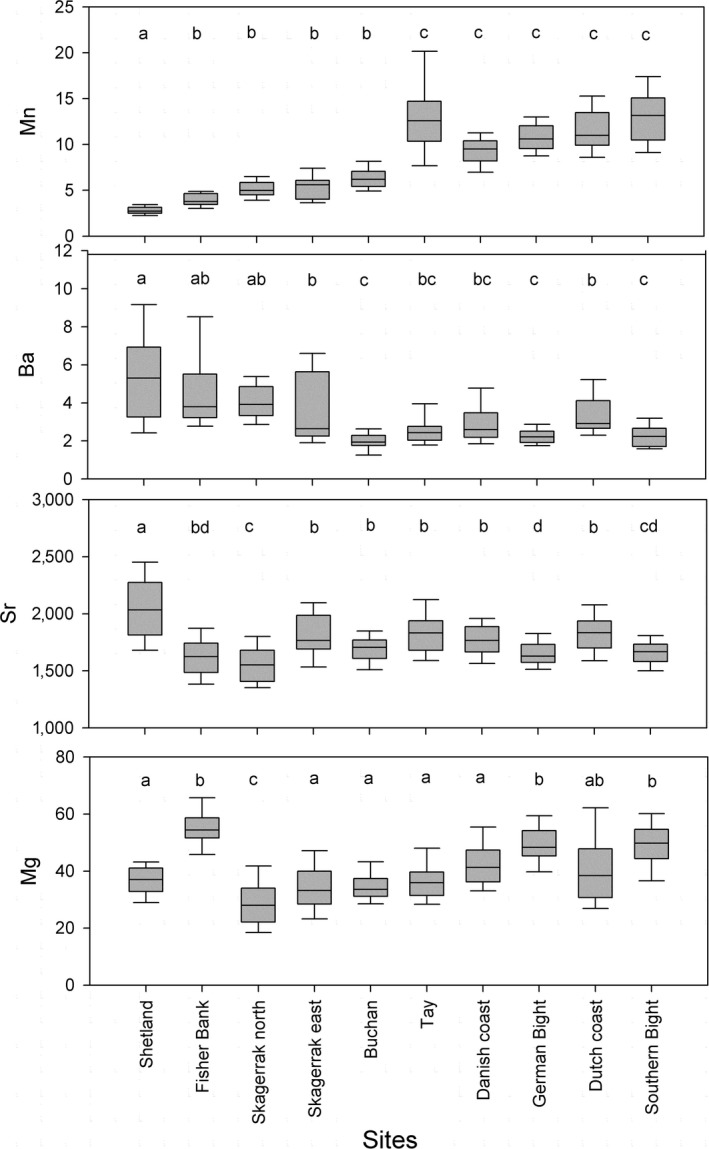
Box plots showing elemental variation, from juvenile WS‐ICP‐MS, for Mn, Ba, Sr, and Mg. The black solid line corresponds to the median, the top and bottom of the box are the 75th and 25th percentiles, while the whiskers show the 5% and 95% percentiles, respectively. Different letters refer to significant differences between sites based on Dunn's multiple comparison test (*p* < 0.05)

Whole juvenile otoliths showed significant geographic variation for Mn (*H* = 373.2, 9 *df*,* p* < 0.0001), Ba (*H* = 226.7, 9 *df*,* p* < 0.0001), Sr (*H* = 154.6, 9 *df*,* p* < 0.0001), and Mg (*H* = 225.5, 9 *df*,* p* < 0.0001). There were significant differences between sites (Bonferroni's test; *p* < 0.001; Figure 4), with the northern sites Shetland and Fisher Bank having high Ba and low Mn, and the southern sites Southern Bight, German Bight, and Danish coast having high Mn.

Differences in whole‐solution juvenile chemistry among sites were reflected in the RF assignment. Classification accuracy by station ranged from 0.25 to 0.87, although individuals were classified to the northern North Sea or southern North Sea to an accuracy of >0.83, as most misassignments were to nearby grounds (Table 4). Classification accuracy was only 0.59 for the Scottish coastal ground of Buchan due to some similarity in elemental composition with east Skagerrak. Similarly, east Skagerrak also had 0.13 assigned to Buchan, as well as 0.08 and 0.13 assigned to southern and northern sites, respectively. However, classification error was far lower in the north Skagerrak site, with none assigned to Buchan and only 0.06 assigned to both southern and northern sites. Based on the classification error, there is a low likelihood of misassigning southern‐to‐northern juvenile sites, but less certainty with assignments to east Skagerrak and Buchan.

**Table 4:** Cross‐validated classification probabilities of whole juvenile chemistry for 10 juvenile sites calculated on 10 independent samples of 80 individuals. Different shaded values refer to classification probability for sites in the same region, and the sum classification probability for these regions is also given

Region	Northern		Skagerrak		East coast		Southern				Region Sum
Site	SH	FB	NS	ES	BU	TA	DA	GB	GC	SB	
SH	0.87	0.07	0.01	0.04	0.00	0.01	0.00	0.00	0.00	0.00	0.94
FB	0.10	0.76	0.07	0.06	0.00	0.00	0.00	0.00	0.00	0.00	0.87
NS	0.01	0.01	0.87	0.05	0.03	0.01	0.01	0.00	0.01	0.00	0.92
ES	0.02	0.11	0.28	0.36	0.15	0.02	0.05	0.00	0.01	0.00	0.64
BU	0.00	0.00	0.08	0.11	0.59	0.08	0.09	0.02	0.02	0.01	0.67
TA	0.00	0.00	0.01	0.02	0.12	0.39	0.09	0.06	0.20	0.10	0.51/0.84^a^
DA	0.00	0.00	0.05	0.02	0.09	0.12	0.25	0.19	0.24	0.05	0.72
GB	0.00	0.00	0.00	0.00	0.03	0.05	0.16	0.49	0.11	0.16	0.92
GC	0.00	0.00	0.00	0.02	0.03	0.19	0.17	0.09	0.38	0.12	0.76
SB	0.00	0.00	0.00	0.00	0.02	0.18	0.09	0.11	0.15	0.45	0.80

Regional classification for Tay considered for Scottish east coast and southern sites (^a^), respectively.

### 3.4 Adult assignment

**Figure 5:**
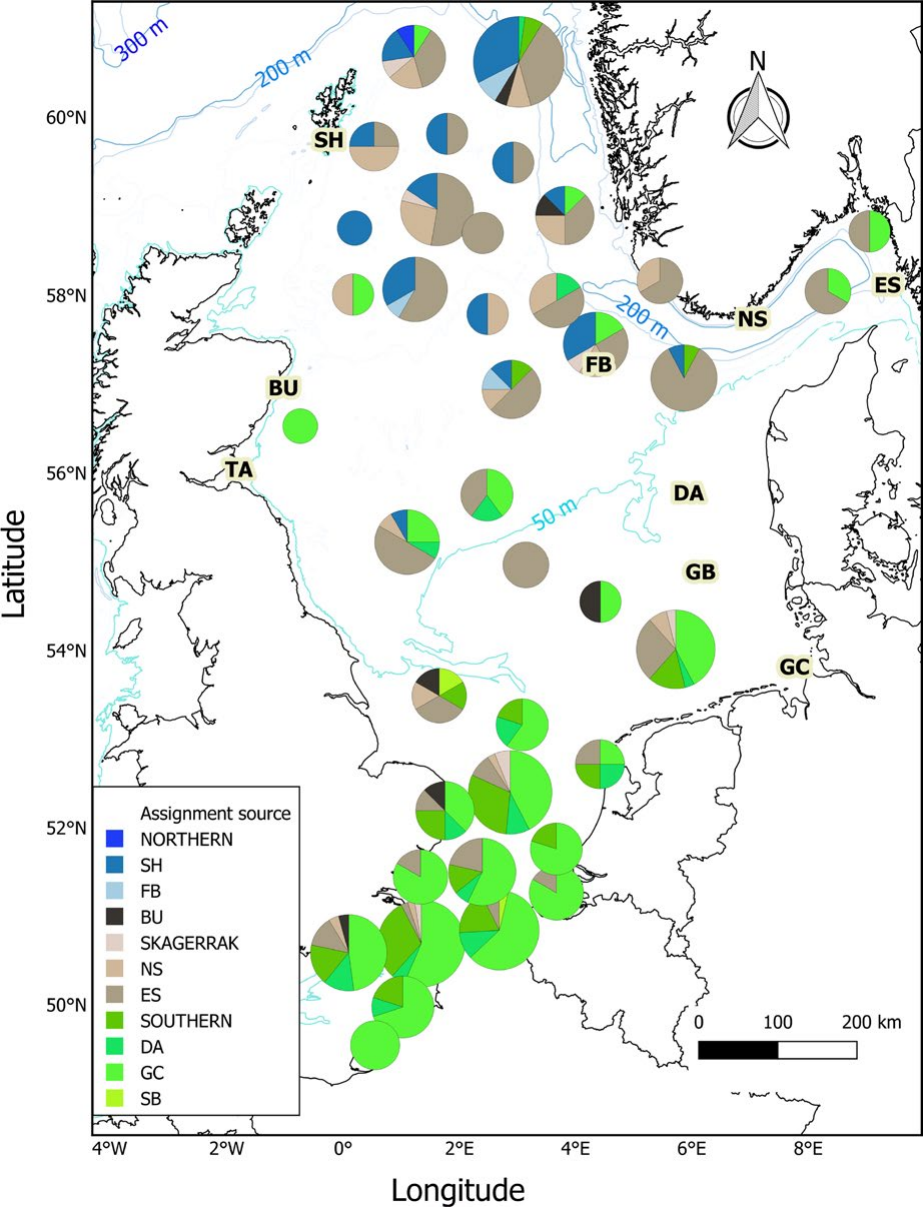
Chart showing assignment of adults to juvenile sites. Pie chart color represents juvenile site assignment, and the size is scaled to the square root of number sampled (maximum *n* = 46). The depth contours are also shown

Results of the Bayesian assignment of the juvenile component of adult otoliths in 2009 and 2010 to juvenile settlement areas are presented in Figure 5, presented as pie charts. These results can be viewed in juxtaposition with the adult density distribution (Figure 1b), which shows that there were two major aggregations in the northeastern and southern North Sea. Of the 442 adults analyzed, 79% had an assignment of >0.5 to a single juvenile ground, and using the same criteria, a further 13% could be assigned to southern, northern, or Skagerrak grounds. Only 5% of adults were outliers that could not be assigned to any site. Almost all adults in the eastern channel and southern North Sea had elemental values consistent with juveniles sampled in the southeastern North Sea, predominantly from the German coast where high densities of juveniles were found in 2007. A significant proportion of northern adult aggregations appeared to originate from juveniles sampled in the northern sites of Shetland and Fisher Bank. However, the Skagerrak appeared to be the major source of recruits to spawning grounds in the northern North Sea. Skagerrak juveniles also appeared to contribute to the central and, to a lesser extent, southern North Sea adult aggregations. Adults in the Skagerrak were either of local juvenile origin or from the German coast.

## 4 DISCUSSION

Through a combination of clustering of near‐core ablation chemistry to consider natal sources and assignment of the juvenile component of adult otoliths to baseline nursery sites, the present study provides a framework for examining how population structure is maintained in species with more than a single dispersive life stage. The approach is dependent on spatial variation in otolith elemental composition (Elsdon et al., 2008) and the assumption that differences in elemental values near the core reflect multiple larval origins (Calò et al., 2016). The elements Mn and Ba gave the most discriminant accuracy and exhibited a latitudinal gradient that provided a natural tag for northern and southern sites. Smaller‐scale differences in Sr and Mg allowed a further degree of separation between Skagerrak and other sites, fulfilling the requirement for spatial variation in elemental values. The observed scale of variation in whole‐solution chemistry was consistent with smaller‐scale studies in cod and other gadoids from the North Sea (Gibb et al., 2007; Tobin, Wright, Gibb, & Gibb, 2010; Wright, Tobin, Gibb, & Gibb, 2010; Wright, Neat, et al., 2006). Although the observed differences in elemental values cannot be explained, variation in water elemental concentrations has been reported over similar spatial scales (e.g., Balls, Cofino, Schmidt, Topping, & Wilson, 1993), and there are oxygen, salinity, and temperature gradients (Berx & Hughes, 2009; Queste, Fernand, Jickells, & Heywood, 2013) that may account for the observed spatial variation in some of the considered elements (Bath et al., 2000). For example, the regional importance of Atlantic inflow in the north and freshwater from the Baltic and major rivers in the south persists all year around (Munk et al., 2002, 2009; Winther & Johannessen, 2006). The two key elements important in discrimination in this study, Mn and Ba, have often found to be reflective of the physicochemical environment (Bath et al., 2000; Thomas, Ganio, Roberts, Stephen, & Swearer, 2017). Seawater concentration of Mn can exhibit an inverse relationship with salinity (Balls et al., 1993), which is consistent with the higher concentrations of this element in both water (Dehairs, Baeyens, & Van Gansbeke, 1989) and otoliths from the south and east of the study area. Ba concentration tends to increase with depth (IPCS 1990), which is consistent with the highest concentrations of otolith Ba from the northern sites that have a strong Atlantic influence. Importantly, the gradients in otolith elemental values enabled distinction of the northern waters that adults from the Viking genetic unit inhabit (Heath et al., 2014), as well as exploration of the finer‐scale structuring suggested by adult movements (Neat et al., 2014; Righton et al., 2007; Wright, Neat, et al., 2006; Wright, Galley, et al., 2006).

As natal clusters were not obtained from sampled larvae, we do not know whether similarities in elemental values between edge and near‐core ablation chemistry reflect a similar spatial distribution of larvae and juveniles. Spawning grounds tend to occur further offshore than the areas where cod settle as juveniles (Gibb et al., 2007; Gonzalez‐Irusta & Wright, 2016), and because of freshwater input into shallow coastal areas, the chemical landscape of settling cod may be expected to be more varied than that experienced by hatching larvae. This may be one explanation for the narrower range in near‐core chemistry compared to edge chemistry. However, different element signatures between life stages may develop due to ontogenetic variation in element incorporation (De Pontual, Lagardère, Amara, Bohn, & Ogor, 2003). In addition to possible ontogenetic effects, elements affected by temperature should not be directly comparable between the near‐core and edge chemistry because they were formed around 11–19°C at the edge and around 7°C near the core, based on the temperature during spawning (Gonzalez‐Irusta & Wright, 2016). Stanley et al. (2015) found that otoliths of juvenile cod had around a third higher Mn and Mg in individuals held at 12°C compared to 5°C. Consequently, the lower near‐core values of these elements in our study may be partially explained by the difference in temperature. However, the threefold variation in median Mn and Mg values among sites suggests that spatial differences would be far more important than temperature in explaining the observed core and edge differences. Variation in salinity across the study area (33.21–34.5) was also too low to expect that this variable had any significant effect when considered in the context of the study by Stanley et al. (2015).

Clustering of near‐core chemistry indicated more than one contributing larval source, consistent with genetic evidence (Heath et al., 2014; Nielsen et al., 2009; Poulsen et al., 2011). The Southern Bight site was almost exclusively composed of individuals belonging to natal cluster A, characterized by high Mn and Mg but low Ba, similar to the signature observed in the ablated edge chemistry and the whole juvenile signature of Southern Bight individuals. While natal cluster A seems to also contribute to the Danish coast site and Buchan, cluster B, also characterized by high Mn and low Ba, is a major contributor to sites of the central North Sea, from Buchan to the Danish coast. While minor contributions are found in sites of the central North Sea (Danish coast, German Bight, Buchan, and Tay), cluster C is a major contributor to the two Skagerrak sites and Fisher bank. It is characterized by low Ba, Mn, and Mg. Most northern sites had a cluster D signal associated with low Mn and high Ba, similar to the edge and whole otolith signatures of the northern juvenile sites. The low proportion of individuals assigned to cluster D in other sample locations suggests a largely separate natal source. While it is not directly possible to assign a location to these natal clusters, the observed similarities between the edge signature and that of the near‐core clusters indicate that the processes (whether genetic, physiological, or environmental) involved in generating the observed spatial variation in otolith chemistry are similar between the larval and juvenile stages.

The evidence for segregated larval sources between north and south is inconsistent with a common propagule pool of larvae. The scale of similarities between near‐core clusters and juvenile edge chemistry, as well as between the juvenile component of adults and whole juvenile chemistry, was largely consistent with the spatial extent of the deep northern and shallow southern population units proposed by Heath et al. (2014) and evident from tagging (Neat et al., 2014; Wright, Neat, et al., 2006). While this appears suggestive of natal fidelity within the boundaries of proposed population units, inferred movements appeared more complex in the northern Skagerrak, as many juveniles had near‐core chemistry that differed significantly from the edge, and many adults in the North Sea had a juvenile Skagerrak signature.

The segregation of near‐core cluster and edge chemistry between juveniles north and south of the 50‐m‐deep contour was consistent with predictions from a biophysical model (Heath et al., 2008). In the southern North Sea, spawning is concentrated in coastal waters (Gonzalez‐Irusta & Wright, 2016) and egg and larval dispersal tends to be confined by adjacent recurrent salinity fronts (Munk et al., 2002, 2009). The similarity in juvenile ablated edge and near‐core chemistry from southern sites therefore appears consistent with the degree of hydrographic retention in this region. Following settlement, juvenile cod tend to exhibit a degree of site fidelity until 1‐year‐old (Hawkins, Soofiani, & Smith, 1985; Riley & Parnell, 1984). Tagging studies also indicate that the southern extent of the Viking unit and northern extent of the Dogger unit do not overlap (Neat et al., 2014; Righton et al., 2007; Wright, Galley, et al., 2006). This regional fidelity could explain why most adults in the south were assigned to nearby juvenile sites. Consequently, the Dogger unit in the southern and central North Sea appears to be maintained by a combination of hydrographic retention of larvae and regional fidelity of the settled juvenile and adult cod.

The similarity between juvenile edge and near‐core chemistry seen in Buchan off the Scottish east coast, together with past evidence for a local nursery origin of spawning cod (Wright, Neat, et al., 2006), appears consistent with an even finer spatial scale of natal fidelity than that seen in the southern North Sea. High densities of juvenile cod do occur in the coastal waters of this region (Gibb et al., 2007), which are in close proximity to spawning grounds (Gonzalez‐Irusta & Wright, 2016). Unfortunately, too few adults were caught close to the Scottish east coast to verify fidelity during this study. However, given the similarity in juvenile natal and edge values, it is unlikely that past evidence of the links between adults and nearby juvenile sites (Wright, Neat, et al., 2006) can be explained by the adopted migrant hypothesis (McQuinn, 1997; Petitgas et al., 2010), as such juveniles would be expected to have had a different natal signature than just the region they settled to. Moreover, differences in maturation schedules between this region and the southern North Sea, based on field investigations (Wright et al., 2011) and common environment experiments (Harrald, Wright, & Neat, 2010), are suggestive of local adaptation within a reproductively isolated population.

Patterns of overlap in the chemical signatures between the juvenile component of northern North Sea adults and juveniles from the northern Skagerrak site appear consistent with natal homing. As with juveniles in the northern North Sea, a high proportion of northern Skagerrak juveniles had high Ba near the core, contrary to the corresponding edge chemistry. Advection of larvae into the Skagerrak is quite likely as southwesterly winds dominate during the spawning season of cod, which tends to take eggs and larvae south and east (Winther & Johannessen, 2006) and often into the Skagerrak (Jonsson et al., 2016). The high assignment of northern North Sea spawners to the Skagerrak juvenile signature suggests that the latter area acts as a nursery and could explain why densities of this life stage are so low in the former region. Previous genetic, otolith microchemistry and archival tagging studies in the Skagerrak have demonstrated that there is a mix of resident and nonresident juvenile cod, with a shift in the latter toward the North Sea by around age 2 to 3 (Knutsen et al., 2004; Svedäng, Righton, & Jonsson, 2007; Svedäng et al., 2010). The present study helps explain these earlier findings, as most sampled juveniles in the northern Skagerrak appeared to belong to the deep northern North Sea “Viking” unit. Once recruited to this Viking region, electronic tagging records suggest that cod remain in depths >100 m all year around and experience a much smaller temperature range than the Dogger population unit (Neat et al., 2014). Juvenile cod from the Northern Skagerrak site have also been found to largely originate from the North Sea (Barth et al., 2017). While future genetic investigation is needed to confirm the links between northern and southern North Sea spawning grounds, this apparent return migration is suggestive of natal philopatry.

Knowledge of life‐stage connectivity is clearly important in understanding the changes in population dynamics. In the case of cod, past studies have treated the North Sea as a single unit and correlated the center of distribution with environmental conditions (e.g., Rindorf & Lewy, 2006; Rutterford et al., 2015). However, this study suggests that such a treatment could fail to account for important differences in substock dynamics. Importantly, this study demonstrates that juveniles move both northward and southward within a year‐class, dependent on the nursery area they settle to and their region of origin. Hence, reported northern shifts in North Sea stock distribution are more likely to reflect higher population growth in the Viking than in the Dogger unit. Indeed, accounting for such structuring, Holmes, Millar, Fryer, and Wright (2014) found evidence that the Dogger spawning stock biomass had undergone a much greater decline than that seen in the Viking unit. The links between spawning units and juveniles evident from this study now make it possible to consider the relationships between recruitment and spawning stock at a population level.

The challenge of characterizing population structuring in fully marine species is evident from the paucity of published studies. Indeed, the question of natal homing in cod has long been debated (Barth et al., 2017; Svedäng et al., 2007, 2010) with the only convincing genetic evidence being recently reported (Barth et al., 2017; Bonanomi et al., 2016). While genetic approaches allow for more generalized conclusions about multigeneration philopatry, they can be biased by rare mixing events and the evolutionary timescales over which genetic drift occurs. Genetic differences may also provide little insight into ecologically relevant connectivity (i.e. levels sufficient to influence demographic rates), because only a few individuals need to be exchanged per generation to maintain genetic homogeneity between subpopulations (Waples & Gaggiotti, 2006; Wright, 1931). Natural chemical tags, such as those used in this study, provide a contemporary indication of movements between life stages and could be used in combination with genetic tools to improve the understanding of the role of natal philopatry in structuring marine fish populations.

## CONFLICT OF INTEREST

None declared.

## AUTHOR CONTRIBUTIONS

PW conceived the ideas and led the writing of the manuscript; PW and TR designed the methodology and analyzed the data. JA, FG, and SD analyzed samples. All authors contributed critically to the drafts and gave the final approval for publication.

Data are available from Marine Scotland DATA http://data.marine.gov.scot.

## References

[ece34186-bib-0001] Balls, P. , Cofino, W. , Schmidt, D. , Topping, G. , & Wilson, S. (1993). ICES baseline survey of trace metals in European shelf waters. ICES Journal of Marine Science, 50, 435–444. 10.1006/jmsc.1993.1047

[ece34186-bib-0002] Barnes, T. C. , Gillanders, B. M. , & Rose, K. (2013). Combined effects of extrinsic and intrinsic factors on otolith chemistry: Implications for environmental reconstructions. Canadian Journal of Fisheries and Aquatic Sciences, 70, 1159–1166. 10.1139/cjfas-2012-0442

[ece34186-bib-0003] Barth, J. M. I. , Berg, P. R. , Jonsson, P. R. , Bonanomi, S. , Corell, H. , Hemmer‐Hansen, J. , … André, C. (2017). Genome architecture enables local adaptation of Atlantic cod despite high connectivity. Molecular Ecology, 26, 4452–4466. 10.1111/mec.14207 28626905

[ece34186-bib-0004] Bastrikin, D. K. , Gallego, A. , Millar, C. P. , Priede, I. G. , & Jones, E. G. (2014). Settlement length and temporal settlement patterns of juvenile cod (*Gadus morhua*), haddock (*Melanogrammus aeglefinus*), and whiting (*Merlangius merlangus*) in a northern North Sea coastal nursery area. ICES Journal of Marine Science, 71, 2101–2113. 10.1093/icesjms/fsu029

[ece34186-bib-0005] Bath, G. E. , Thorrold, S. R. , Jones, C. M. , Campana, S. E. , McLaren, J. W. , & Lam, J. W. H. (2000). Strontium and barium uptake in aragonitic otoliths of marine fish. Geochimica et Cosmochimica Acta, 64, 1705–1714. 10.1016/S0016-7037(99)00419-6

[ece34186-bib-0006] Berx, B. , & Hughes, S. L. (2009). Climatology of surface and near‐bed temperature and salinity on the north‐west European continental shelf for 1971–2000. Continental Shelf Research, 29, 2286–2292. 10.1016/j.csr.2009.09.006

[ece34186-bib-0007] Bonanomi, S. , Overgaard Therkildsen, N. , Retzel, A. , Berg Hedeholm, R. , Pedersen, M. W. , Meldrup, D. , … Nielsen, E. E. (2016). Historical DNA documents long‐distance natal homing in marine fish. Molecular Ecology, 25, 2727–2734. 10.1111/mec.13580 26859133

[ece34186-bib-0008] Bradbury, I. R. , Hubert, S. , Higgins, B. , Bowman, S. , Borza, T. , Paterson, I. G. , … Bentzen, P. (2013). Genomic islands of divergence and their consequences for the resolution of spatial structure in an exploited marine fish. Evolutionary Applications, 6, 450–461. 10.1111/eva.12026 23745137 PMC3673473

[ece34186-bib-0009] Bradbury, I. R. , & Laurel, B. J. (2007). Defining ‘natal homing’ in marine fish populations: Comment on Svedäng et al. (2007). Marine Ecology Progress Series, 349, 307–308. 10.3354/meps07281

[ece34186-bib-0010] Breiman L. (2001). Random forests. Machine Learning, 45, 5–32. 10.1023/A:1010933404324

[ece34186-bib-0011] Calò, A. , Di Franco, A. , De Benedetto, G. E. , Pennetta, A. , Pérez‐Ruzafa, Á. , & García‐Charton, J. A. (2016). Propagule dispersal and larval patch cohesiveness in a Mediterranean coastal fish. Marine Ecology Progress Series, 544, 213–224. 10.3354/meps11609

[ece34186-bib-0012] Campana, S. E. , & Thorrold, S. R. (2001). Otoliths, increments, and elements: Keys to a comprehensive understanding of fish populations? Canadian Journal of Fisheries and Aquatic Sciences, 58, 30–38. 10.1139/f00-177

[ece34186-bib-0013] Charlier, B. , Ginibre, C. , Morgan, D. , Nowell, G. , Pearson, D. , Davidson, J. , & Ottley, C. (2006). Methods for the microsampling and high‐precision analysis of strontium and rubidium isotopes at single crystal scale for petrological and geochronological applications. Chemical Geology, 232, 114–133. 10.1016/j.chemgeo.2006.02.015

[ece34186-bib-0014] Clarke, L. M. , Thorrold, S. R. , & Conover, D. O. (2011). Population differences in otolith chemistry have a genetic basis in *Menidia menidia* . Canadian Journal of Fisheries and Aquatic Sciences, 68, 105–114. 10.1139/F10-147

[ece34186-bib-0015] De Pontual, H. , Lagardère, F. , Amara, R. , Bohn, M. , & Ogor, A. (2003). Influence of ontogenetic and environmental changes in the otolith microchemistry of juvenile sole (*Solea solea*). Journal of Sea Research, 50, 199–211. 10.1016/S1385-1101(03)00080-7

[ece34186-bib-0016] Dehairs, F. , Baeyens, W. , & Van Gansbeke, D. (1989). Tight coupling between enrichment of iron and manganese in North Sea suspended matter and sedimentary redox processes: Evidence for seasonal variability. Estuarine, Coastal and Shelf Science, 29, 457–471. 10.1016/0272-7714(89)90080-2

[ece34186-bib-0017] Elsdon, T. S. , Wells, B. K. , Campana, S. E. , Gillanders, B. M. , Jones, C. M. , Limburg, K. E. , … Walther, B. D. (2008). Otolith chemistry to describe movements and life‐history parameters of fishes: Hypotheses, assumptions, limitations and inferences. Oceanography and Marine Biology: An Annual Review, 46, 297–330.

[ece34186-bib-0018] Gibb, F. M. , Gibb, I. M. , & Wright, P. J. (2007). Isolation of Atlantic cod (*Gadus morhua*) nursery areas. Marine Biology, 151, 1185–1194. 10.1007/s00227-006-0565-0

[ece34186-bib-0019] Gibb, F. , Regnier, T. , Donald, K. , & Wright, P. (2017). Sandeel connectivity inferred from otolith microchemistry. Journal of Sea Research, 119, 8–16. 10.1016/j.seares.2016.10.003

[ece34186-bib-0020] Gillanders, B. M. (2002). Temporal and spatial variability in elemental composition of otoliths: Implications for determining stock identity and connectivity of populations. Canadian Journal of Fisheries and Aquatic Sciences, 59, 669–679. 10.1139/f02-040

[ece34186-bib-0021] Gonzalez‐Irusta, J. , & Wright, P. J. (2016). Spawning grounds of Atlantic cod (*Gadus morhua*) in the North Sea. ICES Journal of Marine Science, 73, 304–315. 10.1093/icesjms/fsv180

[ece34186-bib-0022] Harrald, M. , Wright, P. J. , & Neat, F. C. (2010). Substock variation in reproductive traits in North Sea cod (*Gadus morhua*). Canadian Journal of Fisheries and Aquatic Sciences, 67, 866–876. 10.1139/F10-030

[ece34186-bib-0023] Hawkins, A. D. , Soofiani, N. M. , & Smith, G. W. (1985). Growth and feeding of juvenile cod (*Gadus morhua* L.). ICES Journal of Marine Science, 42, 11–32. 10.1093/icesjms/42.1.11

[ece34186-bib-0024] Heath, M. R. , Culling, M. A. , Crozier, W. W. , Fox, C. J. , Gurney, W. S. C. , Hutchinson, W. F. , … Carvalho, G. R. (2014). Combination of genetics and spatial modelling highlights the sensitivity of cod (*Gadus morhua*) population diversity in the North Sea to distributions of fishing. ICES Journal of Marine Science, 71, 794–807. 10.1093/icesjms/fst185

[ece34186-bib-0025] Heath, M. R. , Kunzlik, P. A. , Gallego, A. , Holmes, S. J. , & Wright, P. J. (2008). A model of meta‐population dynamics for North Sea and West of Scotland cod—The dynamic consequences of natal fidelity. Fisheries Research, 93, 92–116. 10.1016/j.fishres.2008.02.014

[ece34186-bib-0026] Holmes, S. , Millar, C. , Fryer, R. , & Wright, P. (2014). Gadoid dynamics: Differing perceptions when contrasting stock versus population trends and its implications to management. ICES Journal of Marine Science, 71, 1433–1442. 10.1093/icesjms/fsu075

[ece34186-bib-0027] Hutchinson, W. F. , Carvalho, G. R. , & Rogers, S. I. (2001). Marked genetic structuring in localised spawning populations of cod *Gadus morhua* in the North Sea and adjoining waters as revealed by microsatellites. Marine Ecology Progress Series, 223, 251–260. 10.3354/meps223251

[ece34186-bib-0028] IPCS (1990). Barium international programme on chemical safety: Environmental health criteria 107. Geneva, Switzerland: World Health Organisation.

[ece34186-bib-0029] Jonsson, P. R. , Corell, H. , André, C. , Svedäng, H. , & Moksnes, P. O. (2016). Recent decline in cod stocks in the North Sea–Skagerrak–Kattegat shifts the sources of larval supply. Fisheries Oceanography, 25, 210–228. 10.1111/fog.12146

[ece34186-bib-0030] Knutsen, H. , André, C. , Jorde, P. E. , Skogen, M. D. , Thuróczy, E. , & Stenseth, N. C. (2004). Transport of North Sea cod larvae into the Skagerrak coastal populations. Proceedings of the Royal Society of London B: Biological Sciences, 271, 1337–1344. 10.1098/rspb.2004.2721 PMC1691739 15306331

[ece34186-bib-0031] Longerich, H. P. , Jackson, S. E. , & Günther, D. (1996). Laser ablation inductively coupled plasma mass spectrometric transient signal data acquisition and analyte concentration calculation. Journal of Analytical Atomic Spectrometry, 11, 899–904. 10.1039/JA9961100899

[ece34186-bib-0032] McQuinn, I. H. (1997). Metapopulations and the Atlantic herring. Reviews in Fish Biology and Fisheries, 7, 297–329. 10.1023/A:1018491828875

[ece34186-bib-0033] Miller, A. , Versace, V. , Matthews, T. , Montgomery, S. , & Bowie, K. (2013). Ocean currents influence the genetic structure of an intertidal mollusc in southeastern Australia—Implications for predicting the movement of passive dispersers across a marine biogeographic barrier. Ecology and Evolution, 3, 1248–1261. 10.1002/ece3.535 23762511 PMC3678479

[ece34186-bib-0034] Munch, S. , & Clarke, L. (2008). A Bayesian approach to identifying mixtures from otolith chemistry data. Canadian Journal of Fisheries and Aquatic Sciences, 65, 2742–2751. 10.1139/F08-169

[ece34186-bib-0035] Munk, P. , Fox, C. J. , Bolle, L. J. , Damme, V. , Cindy, J. , Fossum, P. , & Kraus, G. (2009). Spawning of North Sea fishes linked to hydrographic features. Fisheries Oceanography, 18, 458–469. 10.1111/j.1365-2419.2009.00525.x

[ece34186-bib-0036] Munk, P. , Wright, P. J. , & Pihl, N. J. (2002). Distribution of the early larval stages of cod, plaice and lesser sandeel across haline fronts in the North Sea. Estuarine, Coastal and Shelf Science, 55, 139–149. 10.1006/ecss.2001.0892

[ece34186-bib-0037] Neat, F. , Bendall, V. , Berx, B. , Wright, P. , Cuaig, M. Ó. , Townhill, C. , … Righton, D. A. (2014). Movement of Atlantic cod around the British Isles. Journal of Applied Ecology, 51, 1564–1574. 10.1111/1365-2664.12343

[ece34186-bib-0038] Neuenfeldt, S. , Righton, D. , Neat, F. , Wright, P. , Svedäng, H. , Michalsen, K. , … Metcalfe, J. (2013). Migrations of Atlantic cod in the NE Atlantic: Then, now and the future. Journal of Fish Biology, 82, 741–763. 10.1111/jfb.12043 23464542

[ece34186-bib-0039] Nielsen, E. E. , Wright, P. J. , Hemmer‐Hansen, J. , Poulsen, N. A. , Monro Gibb, I. , & Meldrup, D. (2009). Microgeographical population structure of cod *Gadus morhua* in the North Sea and west of Scotland: The role of sampling loci and individuals. Marine Ecology Progress Series, 376, 213–225. 10.3354/meps07798

[ece34186-bib-0040] OSPAR Commission (2000). Quality status report 2000, Region II – Greater North Sea. London, UK: OSPAR Commission, 136+ xiii pp.

[ece34186-bib-0041] Petitgas, P. , Secor, D. H. , McQuinn, I. , Huse, G. , & Lo, N. (2010). Stock collapses and their recovery: Mechanisms that establish and maintain life‐cycle closure in space and time. ICES Journal of Marine Science, 67, 1841–1848. 10.1093/icesjms/fsq082

[ece34186-bib-0042] Poulsen, N. A. , Hemmer‐Hansen, J. , Loeschcke, V. , Carvalho, G. R. , & Nielsen, E. E. (2011). Microgeographical population structure and adaptation in Atlantic cod *Gadus morhua*: Spatio‐temporal insights from gene‐associated DNA markers. Marine Ecology Progress Series, 436, 231–243. 10.3354/meps09246

[ece34186-bib-0043] Queste, B. Y. , Fernand, L. , Jickells, T. D. , & Heywood, K. J. (2013). Spatial extent and historical context of North Sea oxygen depletion in August 2010. Biogeochemistry, 113, 53–68. 10.1007/s10533-012-9729-9 PMC7175662 32355379

[ece34186-bib-0044] Régnier, T. , Augley, J. , Devalla, S. , Robinson, C. D. , Wright, P. J. , & Neat, F. C. (2017). Otolith chemistry reveals seamount fidelity in a deepwater fish. Deep Sea Research Part I: Oceanographic Research Papers, 121, 183–189. 10.1016/j.dsr.2017.01.010

[ece34186-bib-0045] Righton, D. , Quayle, V. A. , Hetherington, S. , & Burt, G. (2007). Movements and distribution of cod (*Gadus morhua* L.) in the southern North Sea and English Channel: Results from conventional and electronic tagging experiments. Journal of the Marine Biological Association of the United Kingdom, 87, 599–613. 10.1017/S0025315407054641

[ece34186-bib-0046] Riley, J. D. , & Parnell, W. (1984). The distribution of young cod In Dahl E., Danielssen D. S., Moksness E. & Solemdal P. (Eds.), The Propagation of cod Gadus morhua L: An international symposium. Arendal, 14–17 June 1973 (pp. 563–580). Arendal, Norway: Institute of Marine Research, Flodevigen Biological Station.

[ece34186-bib-0047] Rindorf, A. , & Lewy, P. (2006). Warm, windy winters drive cod north and homing of spawners keeps them there. Journal of Applied Ecology, 43, 445–453. 10.1111/j.1365-2664.2006.01161.x

[ece34186-bib-0048] Robinson, C. D. , Devalla, S. , Rompais, M. , & Davies, I. M. (2009). Solution‐based determination of trace elements in biogenic carbonates: Comparison of two sample introduction systems for use in flow injection ICPMS analysis. Journal of Analytical Atomic Spectrometry, 24, 939–943. 10.1039/b904170b

[ece34186-bib-0049] Rutterford, L. A. , Simpson, S. D. , Jennings, S. , Johnson, M. P. , Blanchard, J. L. , Schön, P.‐J. , … Genner, M. J. (2015). Future fish distributions constrained by depth in warming seas. Nature Climate Change, 5, 569–573. 10.1038/nclimate2607

[ece34186-bib-0050] Schindler, D. E. , Hilborn, R. , Chasco, B. , Boatright, C. P. , Quinn, T. P. , Rogers, L. A. , & Webster, M. S. (2010). Population diversity and the portfolio effect in an exploited species. Nature, 465, 609–612. 10.1038/nature09060 20520713

[ece34186-bib-0051] Stanley, R. R. E. , Bradbury, I. R. , DiBacco, C. , Snelgrove, P. V. R. , Thorrold, S. R. , & Killen, S. S. (2015). Environmentally mediated trends in otolith composition of juvenile Atlantic cod (*Gadus morhua*). ICES Journal of Marine Science, 72, 2350–2363. 10.1093/icesjms/fsv070

[ece34186-bib-0052] Svedäng, H. , André, C. , Jonsson, P. , Elfman, M. , & Limburg, K. E. (2010). Migratory behaviour and otolith chemistry suggest fine‐scale sub‐population structure within a genetically homogenous Atlantic Cod population. Environmental Biology of Fishes, 89, 383–397. 10.1007/s10641-010-9669-y

[ece34186-bib-0053] Svedäng, H. , Righton, D. , & Jonsson, P. (2007). Migratory behaviour of Atlantic cod *Gadus morhua*: Natal homing is the prime stock‐separating mechanism. Marine Ecology Progress Series, 345, 1–12. 10.3354/meps07140

[ece34186-bib-0054] Thomas, O. R. B. , Ganio, K. , Roberts, B. R. , Stephen, E. , & Swearer, S. E. (2017). Trace element–protein interactions in endolymph from the inner ear of fish: Implications for environmental reconstructions using fish otolith chemistry. Metallomics, 9, 239–249. 10.1039/C6MT00189K 28091665

[ece34186-bib-0055] Thorisson, K. , Jónsdóttir, I. G. , Marteinsdottir, G. , & Campana, S. E. (2011). The use of otolith chemistry to determine the juvenile source of spawning cod in Icelandic waters. ICES Journal of Marine Science, 68, 98–106. 10.1093/icesjms/fsq133

[ece34186-bib-0056] Thorrold, S. R. , Latkoczy, C. , Swart, P. K. , & Jones, C. M. (2001). Natal homing in a marine fish metapopulation. Science, 291, 297–299. 10.1126/science.291.5502.297 11209078

[ece34186-bib-0057] Tobin, D. , Wright, P. J. , Gibb, F. M. , & Gibb, I. M. (2010). The importance of life stage to population connectivity in whiting (*Merlangius merlangus*) from the northern European shelf. Marine Biology, 157, 1063–1073. 10.1007/s00227-009-1387-7

[ece34186-bib-0058] Turrell, B. W. R. , Henderson, E. W. , Slesser, G. , Payne, R. , & Adams, R. D. (1992). Seasonal changes in the circulation of the northern North Sea. Continental Shelf Research, 12, 257–286. 10.1016/0278-4343(92)90032-F

[ece34186-bib-0059] Walther, B. D. , Kingsford, M. J. , O'Callaghan, M. D. , & McCulloch, M. T. (2010). Interactive effects of ontogeny, food ration and temperature on elemental incorporation in otoliths of a coral reef fish. Environmental Biology of Fishes, 89, 441–451. 10.1007/s10641-010-9661-6

[ece34186-bib-0060] Walther, B. D. , Thorrold, S. R. , & Olney, J. E. (2008). Geochemical signatures in otoliths record natal origins of American shad. Transactions of the American Fisheries Society, 137, 57–69. 10.1577/T07-029.1

[ece34186-bib-0061] Waples, R. S. , & Gaggiotti, O. (2006). What is a population? An empirical evaluation of some genetic methods for identifying the number of gene pools and their degree of connectivity. Molecular Ecology, 15, 1419–1439. 10.1111/j.1365-294X.2006.02890.x 16629801

[ece34186-bib-0062] Winther, N. G. , & Johannessen, J. A. (2006). North Sea circulation: Atlantic inflow and its destination. Journal of Geophysical Research: Oceans, 111, C12018 10.1029/2005JC003310

[ece34186-bib-0063] Wright, S. (1931). Evolution in Mendelian populations. Genetics, 16, 97–159. 17246615 10.1093/genetics/16.2.97 PMC1201091

[ece34186-bib-0064] Wright, P. J. , Galley, E. , Gibb, I. M. , & Neat, F. C. (2006). Fidelity of adult cod to spawning grounds in Scottish waters. Fisheries Research, 77, 148–158. 10.1016/j.fishres.2005.10.008

[ece34186-bib-0065] Wright, P. J. , Millar, C. P. , & Gibb, F. M. (2011). Intrastock differences in maturation schedules of Atlantic cod, *Gadus morhua* . ICES Journal of Marine Science, 68, 1918–1927. 10.1093/icesjms/fsr111

[ece34186-bib-0066] Wright, P. J. , Neat, F. C. , Gibb, F. M. , Gibb, I. M. , & Thordarson, H. (2006). Evidence for metapopulation structuring in cod from the west of Scotland and North Sea. Journal of Fish Biology, 69, 181–199. 10.1111/j.1095-8649.2006.01262.x

[ece34186-bib-0067] Wright, P. J. , Tobin, D. , Gibb, F. M. , & Gibb, I. M. (2010). Assessing nursery contribution to recruitment: Relevance of closed areas to haddock *Melanogrammus aeglefinus* . Marine Ecology Progress Series, 400, 221–232. 10.3354/meps08384

